# The Different Responses of the Soil Fungal and Bacterial Functional Community Structure to Short-Term N and P Additions in an Alpine Steppe on the Tibetan Plateau

**DOI:** 10.3390/biology15161332

**Published:** 2026-08-07

**Authors:** Shaolin Huang, Maoshan Lian, Chengqun Yu, Gang Fu, Wei Sun, Shaowei Li, Fusong Han

**Affiliations:** 1 School of Geography and Tourism, Henan Normal University, Xinxiang 453007, China; huangshaolin@htu.edu.cn; 2 School of Environment, Henan Normal University, Xinxiang 453007, China; lianmaoshan@htu.edu.cn; 3 Lhasa Plateau Ecosystem Research Station, Key Laboratory of Ecosystem Network Observation and Modeling, Institute of Geographic Sciences and Natural Resources Research, Chinese Academy of Sciences, Beijing 100101, China; fugang@igsnrr.ac.cn (G.F.); wsun@igsnrr.ac.cn (W.S.); leesw024@163.com (S.L.); 4 School of Urban and Environment Sciences, Hunan University of Technology, Zhuzhou 412007, China; 15574460321@163.com

**Keywords:** N and P additions, soil microorganism, functional diversity, functional community composition, alpine steppe, Tibetan Plateau

## Abstract

The soil microbial functional community is a better predictor of ecosystem processes under global change. However, the responses of the soil microbial functional community structure to short-term nitrogen (N) and phosphorus (P) additions remain unclear. Here, we carried out an experiment exploring the effects of N and P additions on soil fungal and bacterial functional diversity and community composition in an alpine steppe on the Tibetan Plateau. The results showed that soil fungal functional α-diversity significantly decreased under N or P addition, whereas soil bacterial functional α-diversity did not change. Furthermore, soil bacterial functional group aerobic nitrite oxidation increased significantly under combined N and P additions, but soil bacterial and fungal community composition and β-diversity did not change. Further analysis illustrated that soil available nitrogen was the main factor leading to the changes in soil fungal functional α-diversity and soil bacterial functional groups under N and P additions. Therefore, we should give importance to the potential influence in view of future increasing N and P deposition on the soil microbial functional community in the alpine grassland on the Tibetan Plateau, which will be beneficial for the prediction of soil biogeochemical cycles and guiding ecosystem management.

## Simple Summary

Soil microbial functional communities are vital for nutrient cycling, but their response to short-term nitrogen (N) and phosphorus (P) additions is poorly known. In a Tibetan alpine steppe, we found that N and P additions reduced fungal functional diversity and boosted a bacterial group involved in aerobic nitrite oxidation, while bacterial diversity remained unchanged. These shifts were mainly driven by soil available nitrogen. Our results suggest that increasing N and P deposition could alter microbial functions, potentially affecting soil carbon and nutrient turnover. This highlights the importance of managing nutrient inputs to maintain healthy alpine grassland ecosystems.

## 1. Introduction

As important limiting factors for the structure and function of terrestrial ecosystems such as grasslands, nitrogen (N) and phosphorus (P) are closely related to the maintenance, succession and stability of the ecosystem [1,2]. Since the 20th century, anthropogenic activities such as the combustion of fossil fuels, the application of N and P fertilizers, the development of animal husbandry, and the emission of vehicle exhaust have led to a significant increase in global N and P deposition and thus changed soil conditions in natural ecosystems [3,4]. The changed soil conditions, including total nutrient content [5,6], available nutrients, and so on [5,7], further influence the transformation of soil carbon (C), N, and P cycles [8,9].

Soil microbial communities drive the Earth’s key biogeochemical cycles, maintain vegetation productivity, and adjust the responses of terrestrial ecosystems to global change [10,11]. Soil bacteria and fungi constitute the main components of the soil microbial communities, which play a significant role in facilitating the biogeochemical cycles of elements such as C, N, and P in terrestrial ecosystems [12,13,14]. The dimensions of soil bacterial and fungal diversity encompass species diversity, functional diversity, and phylogenetic diversity [15]. The diversity of soil bacterial and fungal communities is highly sensitive to external disturbances, such as elevated temperatures [16], increased nutrients, changed precipitation, and so on [17]. There are numerous previous studies on the effects of N and P additions on soil bacterial and fungal diversity and community composition, including the species and phylogenetic dimensions [18,19]. Although there have been some earlier studies on the effects of N and P additions on soil bacterial and fungal functional groups in pine forests [5], semiarid grassland [6,20], and alpine grasslands [21], which have reported changes in some of the functional groups, there is currently limited attention on functional diversity [22]. Functional diversity is generally better at predicting ecosystem processes than taxonomic diversity [23,24]. Further analysis shows that soil pH [20], soil moisture [25], and total phosphorus [21] are important factors affecting microbial functional groups due to the differences in addition levels, addition time, climatic conditions, and vegetation types.

The Tibetan Plateau (TP) is known as the “Roof of the World” and the “Third Pole of the Earth” and is an important ecological security barrier for both China and Asia [26]. Due to its high altitude, low temperature, and low air pressure, the ecosystem of the TP is extremely sensitive to global changes [27]. A large number of studies have explored the influence patterns and driving mechanisms of N and P additions on the ecosystem of the high-altitude grassland of the TP. However, these previous studies mainly focused on the plant community structure (species diversity, etc.) and functions (productivity, etc.) in the eastern part of the TP, as well as soil carbon stocks, nutrients (nitrogen, phosphorus, potassium, etc.), soil acidification, and soil microbial community structure (microbial biomass, species diversity, etc.) [28,29,30]. Firstly, there is relatively little research on the structure and function of soil microbial communities in the western part of the TP, but studies have indicated that the ecological environment is more fragile and sensitive in this area [31]. Secondly, in order to assess the direct effects and predominant factors, we establish a short-term N and P fertilization experiment (1 month), similar to Bradley [32]. Therefore, evaluating the functional diversity and community composition of soil bacteria and fungi in response to N and P additions and the drivers is vital for our predicting and understanding ecosystem changes in the context of global change on the TP.

Here, we investigated the responses of soil bacterial and fungal functional diversity and community composition to short-term N and P additions in the alpine steppe on the TP. We primarily explored (1) how the N and P additions can influence the soil bacterial and fungal functional diversity and community composition, and (2) the predominant factor influencing the soil bacterial and fungal functional community structure.

## 2. Materials and Methods

### 2.1. Study Site and Experimental Design

This study was conducted in an alpine steppe of Zhongba County (29°37′43″ N, 82°21′59″ E, 4763 m, Figure S1), Tibet Autonomous Region, China. The mean yearly temperature was about 5.1 °C, and the mean yearly precipitation was about 320 mm. Before the experiment, we determined the vegetation conditions and soil physicochemical properties of the study site. The dominant species in the alpine steppe was *Potentilla bifurca* Linn., with a coverage of 15.75%, accompanied by *Carex moorcroftii* Falc. ex Boott with a coverage of 8.60%, *Kobresia humilis* with a coverage of 6.75%, *Microula sikkimensis* with a coverage of 1.50%, etc. The plant community of the study site is shown in Figure S2. The soil organic carbon, total nitrogen, total phosphorus, and pH were 0.57–0.81%, 0.05–0.08%, 0.02–0.03%, and 8.27–8.47, respectively.

In August 2021, a homogeneous steppe was selected for the N and P fertilization experiment with a two-factor experimental design. The field experiment comprised three N addition rates (0, 5, and 10 g N m^−2^ y^−1^ of urea, Gansu Liuhua Co., Ltd., Baiyin, China) and P (0, 2.5, and 5 g P m^−2^ y^−1^ of superphosphate, Gansu Baiyin Hubao Chemical Co., Ltd., Baiyin, China), resulting in 9 treatments: N0P0 (control), N5, N10, P2.5, P5, N5P2.5, N5P5, N10P2.5, and N10P5. The N and P addition dosages were determined based on the results of earlier experiments in alpine grassland ecosystems [33]. Each treatment included five replicates in 5 m × 5 m plots, totaling 45 plots arranged in a randomized design. To avoid marginal effects from different fertilizers, the distance between the two adjacent plots was about 3 m.

### 2.2. Examination of Vegetation Communities, Soil Sampling, and Analysis

In September 2021, we recorded total coverage within a 0.5 m × 0.5 m quadrat placed at the center of each plot, along with the number of plant species, the height, and coverage of each species. Following the vegetation survey, soil cores (3.8 cm diameter) were collected from the 0 to 10 cm depth in each quadrat. Five soil cores were randomly extracted and then blended to take a composite soil sample in each plot. The composite sample was passed through a 2.0 mm sieve to remove stones and roots. We collected a total of 45 samples. Some of the soil samples were promptly placed in a nitrogen canister for high-throughput sequencing analysis (File S1), and some of the soil samples retained freshness in the refrigerator for assessments of ammonium nitrogen (NH_4_^+^-N), nitrate nitrogen (NO_3_^−^-N), ratio of available nitrogen to available phosphorus (AN:AP), ratio of ammonium nitrogen to nitrate nitrogen (NH_4_^+^-N:NO_3_^−^-N), soil moisture (SM), available phosphorus (AP), soil organic carbon (SOC), total phosphorus (TP), total nitrogen (TN), ratio of carbon to nitrogen (C:N), ratio of carbon to phosphorus (C:P), ratio of nitrogen to phosphorus (N:P), soil sucrase (SC), soil cellulase (CL), soil urease (UA), soil catalase (CT), and soil alkaline phosphatase (ALP).

### 2.3. Statistical Analysis

Two-way analysis of variance (ANOVA) was performed to estimate the effects of N addition, P addition, and their interaction on soil properties, plant diversity, and soil fungal and bacterial functional diversity, followed by Duncan’s test for multiple comparisons. Before the analysis, the Shapiro–Wilk test and Levene’s test were used to test the normality of the distribution and homogeneity of variance, respectively. Spearman correlation analysis between soil fungal and bacterial functional α-diversity and soil and plant variables was carried out.

We used the Microeco package (1.0.0) to obtain the soil fungal and bacterial species α-diversity (OTUs, ACE, Chao1, Shannon’s index, and Simpson’s index) and β-diversity [29]. Based on this, we used FUNGuild and FAPROTAX in the Microeco package to calculate the soil fungal functional α-diversity (Guilds, ACE, Chao1, Shannon’s index, and Simpson’s index) and β-diversity and soil bacterial functional α-diversity (Observed, ACE, Chao1, Shannon’s index, and Simpson’s index) and β-diversity, respectively [34,35]. Regarding soil fungi, there were 298, 373, and 243 OTUs that had confidence rankings of highly probable, probable, and possible, respectively. We only used the 671 OTUs that were ranked with highly probable and probable confidence rankings for the analyses related to ecological functions. We used the Random Forest package (4.6-14) to calculate the relative contribution to soil fungal and bacterial functional α-diversity of every relevant variable [36]. Using nonmetric multidimensional analysis (NMDS), the changes in plant community composition and functional community composition of soil bacteria and fungi for any two treatments of N and P interaction were calculated [36]. We used the Adonis2 significance test to calculate the functional community composition of soil bacteria and fungi. We used the Microeco package to carry out a differential abundance test for soil fungal and bacterial functional groups based on the LEfSe method and then used Duncan’s multiple comparisons to calculate the differential functional groups [5]. The Microeco package was used to perform the Mantel test between the functional community composition of soil fungi and bacteria and environmental variables [5]. The software used in this study included R4.0.2 (Rstudio, Auckland, New Zealand, 2024), SPSS 25 (IBM, Chicago, IL, USA, 2025), and Origin 2026 (OriginLab, Northampton, MA, USA, 2026).

## 3. Results

### 3.1. Variations in Soil and Plant Characteristics Along the N and P Addition Gradient

**Figure 1:**
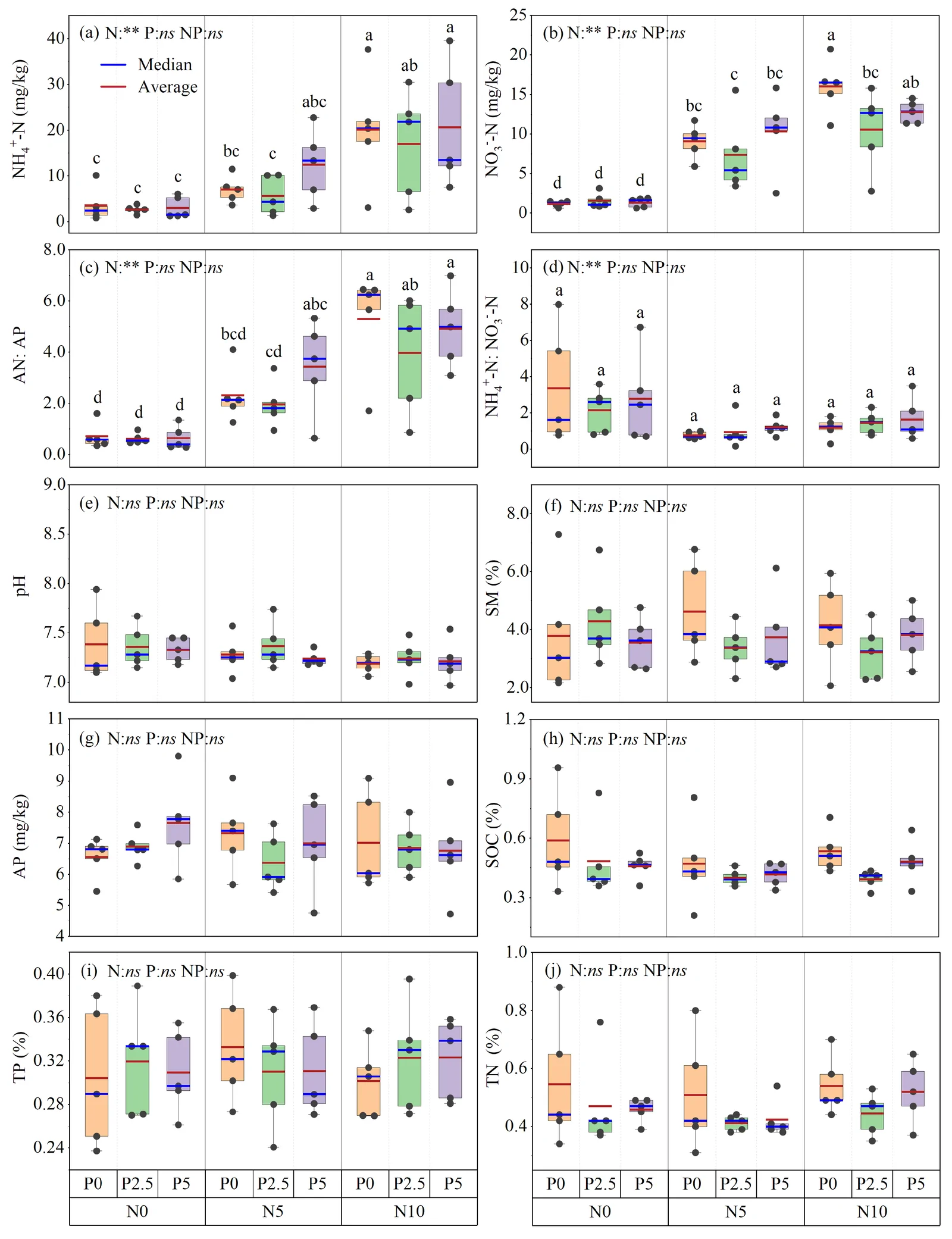
Effects of N and P coupling on ten soil conventional physical and chemical properties (mean ± SD, *n* = 5; ** *p* < 0.01, *ns* indicates non-significant differences). Different letters indicate significant differences among N and P coupling treatments (Duncan’s multiple comparison, *p* < 0.05): (**a**) NH_4_^+^-N: ammonium nitrogen; (**b**) NO_3_^−^-N: nitrate nitrogen; (**c**) AN:AP: ratio of available nitrogen to available phosphorus; (**d**) NH_4_^+^-N:NO_3_^−^-N: ratio of ammonium nitrogen to nitrate nitrogen; (**e**) pH; (**f**) SM: soil moisture; (**g**) AP: available phosphorus; (**h**) SOC: soil organic carbon; (**i**) TP: total phosphorus; (**j**) TN: total nitrogen.

N addition significantly affected soil available nitrogen, including NH_4_^+^-N, NO_3_^−^-N, AN:AP, and NH_4_^+^-N:NO_3_^−^-N (Figure 1a–d). Compared to N0P0, the soil NH_4_^+^-N under N10, N10P2.5, and N10P5 all increased by 5.55-, 4.69-, and 5.69-fold, respectively. Compared to N0P0, the soil NO_3_^−^-N under N5, N10, N5P2.5, N5P5, N10P2.5, and N10P5 all increased by 7.68-, 13.56-, 6.22-, 8.75-, 8.96-, and 10.82-fold, respectively. Compared to N0P0, the soil AN:AP under N10, N5P5, N10P2.5, and N10P5 all increased by 7.46-, 4.84-, 5.58-, and 6.92-fold, respectively. However, N, P, and NP additions did not significantly change other soil characteristics, plant α-diversity, and community composition (Figures S3–S5).

### 3.2. Variations in Soil Fungal and Bacterial Functional α-Diversity Along N and P Additions and Their Relation with Soil and Plant Characteristics

**Figure 2:**
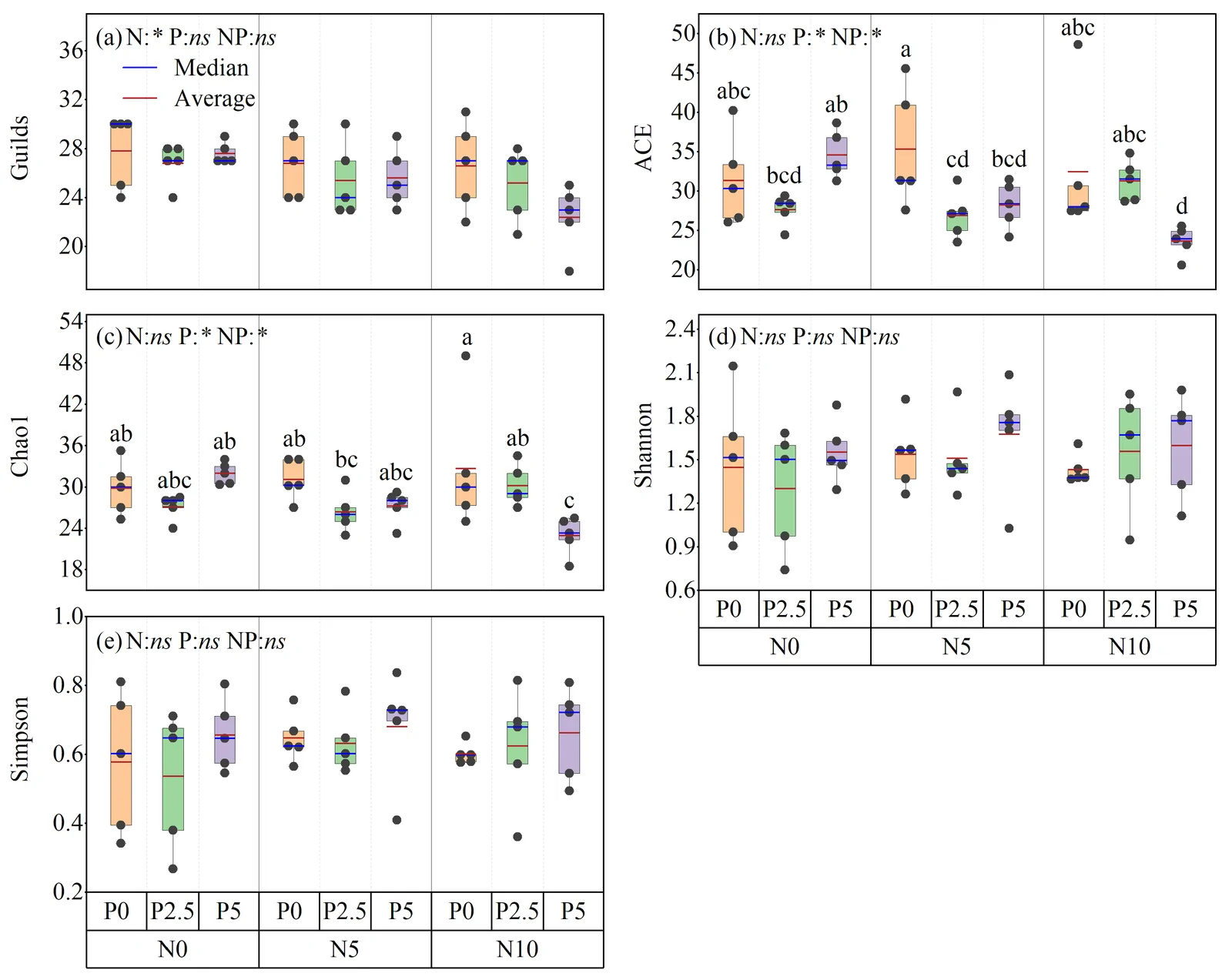
Effects of N and P coupling on soil fungal functional α-diversity in an alpine steppe (mean ± SD, *n* = 5; two-way ANOVA, * *p* < 0.05, *ns* indicates non-significant differences). Different letters indicate significant differences among N and P coupling treatments (Duncan’s multiple comparison, *p* < 0.05): (**a**) Guilds; (**b**) ACE; (**c**) Chao1; (**d**) Shannon; (**e**) Simpson.

N addition significantly changed the soil fungal functional α-diversity Guilds index. P and NP additions significantly changed soil fungal ACE and Chao1 indices. Compared to N0P0, ACE and Chao1 significantly decreased under N10P5 in an alpine steppe (Figure 2). However, N, P, and NP additions did not significantly change soil bacterial functional α-diversity with respect to the Observed, ACE, Chao1, Shannon’s index, and Simpson’s index values (Figure S6).

**Figure 3:**
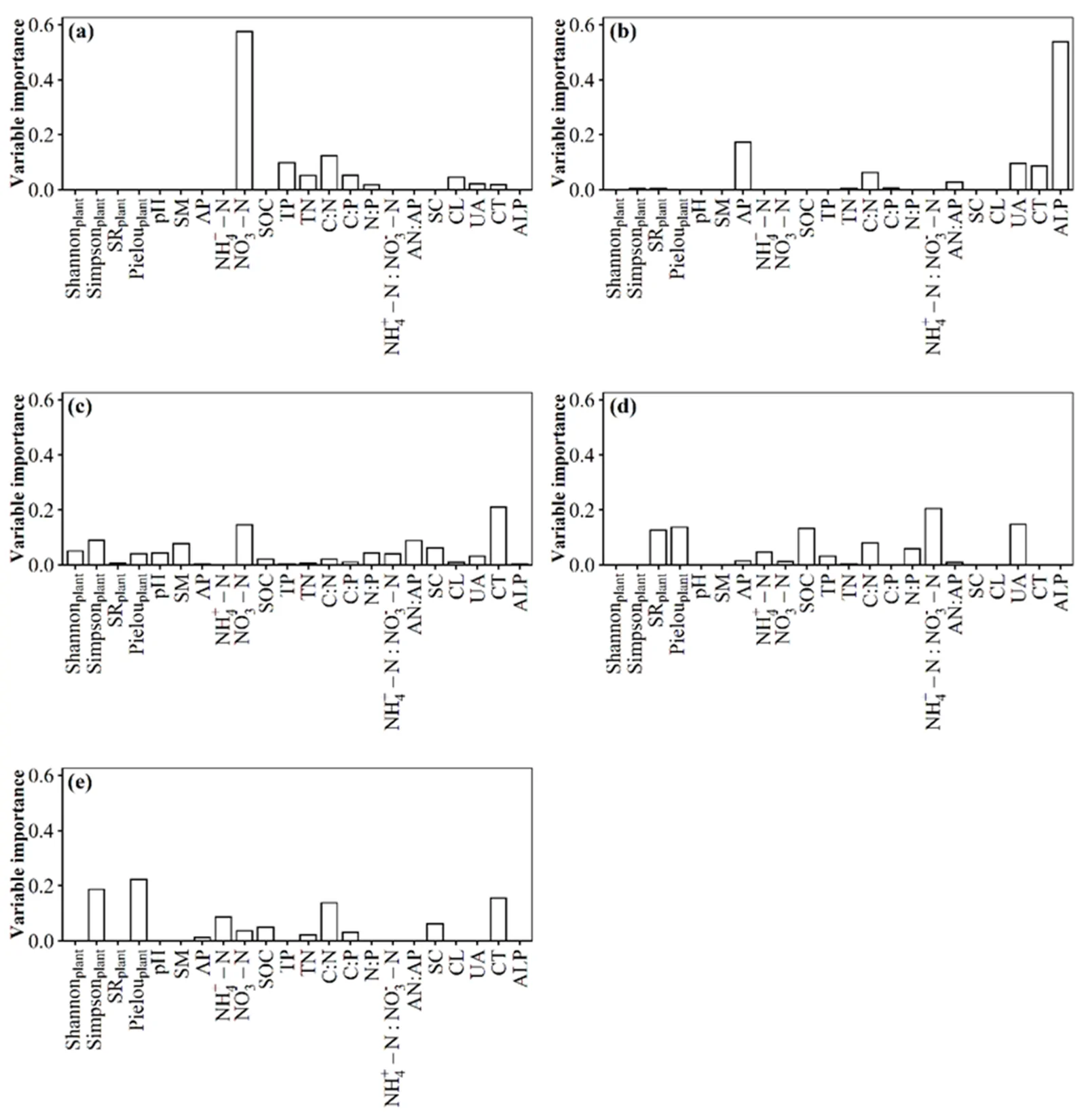
Relative contribution of observed soil and plant variables to soil fungal functional α-diversity, including (**a**) Guilds, (**b**) ACE, (**c**) Chao1, (**d**) Shannon, and (**e**) Simpson to N and P coupling in an alpine steppe.

The relative contributions of environmental variables to the changes in Guilds were NO_3_^−^-N, C:N, TP, C:P, TN, etc., which jointly explained 41.93% of the variation, and Guilds were negatively correlated with TP and SM and positively correlated with C:N. The relative contributions of environmental variables to the changes in ACE were ALP, AP, UA, CT, C:N, AN:AP, etc., which jointly explained 21.1% of the variation, and ACE was negatively correlated with NO_3_^−^-N and AN:AP. The relative contributions of environmental variables to the changes in Chao1 were CT, NO_3_^−^-N, Simpson_plant_, AN:AP, SM, etc., which jointly explained 28.12% of the variation, and Chao1 was negatively correlated with NO_3_^−^-N and AN:AP and positively correlated with CT. The relative contributions of environmental variables to the changes in Shannon’s index were NH_4_^+^-N:NO_3_^−^-N, UA, Pielou_plant_, SOC, SR_plant_, C:N, etc., which jointly explained 12.89% of the variation, and Shannon’s index was negatively correlated with NH_4_^+^-N:NO_3_^−^-N, SM, CT, and UA. The relative contributions of environmental variables to the changes in Simpson’s index were Pielou_plant_, Simpson_plant_, CT, C:N, NH_4_^+^-N, etc., which jointly explained 46.33% of the variation, and Simpson’s index was negatively correlated with SM and CT (Figure 3, Table 1).

The relative contributions of environmental variables to the changes in Observed were SM, UA, NH_4_^+^-N:NO_3_^−^-N, etc., which jointly explained 31.15% of the variation, and Observed was negatively correlated with TP and SC and positively correlated with NH_4_^+^-N and AN:AP. The relative contributions of environmental variables to the changes in ACE were AP, SOC, Simpson_plant_, UA, TN, etc., which jointly explained 41.15% of the variation, and ACE was negatively correlated with TP and SC and positively correlated with NH_4_^+^-N, NO_3_^−^-N, and AN:AP. The relative contributions of environmental variables to the changes in Chao1 were TN, SC, N:P, Shannon_plant_, Simpson_plant_, etc., which jointly explained 41.09% of the variation, and Chao1 was negatively correlated with TP and SC and positively correlated with NH_4_^+^-N and AN:AP. The relative contributions of environmental variables to the changes in Shannon’s index were ALP, SM, C:N, NH_4_^+^-N, etc., which jointly explained 56.55% of the variation, and Shannon’s index was negatively correlated with Shannon_plant_, Simpson_plant_, SOC, C:P, SC, and ALP. The relative contributions of environmental variables to the changes in Simpson’s index were UA, TP, SM, CL, NH_4_^+^-N, etc., which jointly explained 23.63% of the variation, and Simpson’s index was positively correlated with TP and SM (Figure S7, Table S1).

### 3.3. Variations in Soil Fungal and Bacterial Functional Community Composition Along the N and P Addition Gradients and Their Relationship with Soil and Plant Characteristics

Through nonmetric multidimensional analysis (NMDS) based on Bray–Curtis distance and Adonis2 significance testing, it was found that N, P, and NP additions did not alter the functional community composition and β-diversity of soil fungi and bacteria (Figure S8). Soil fungal functional community composition at the N5 level and the N5P2.5 level, the N5 level and the N10P5 level, and the P2.5 level and the N10P5 level showed differences. Soil bacterial functional community composition at the N10 level and the P2.5 level, the P2.5 level and the P5 level, the P2.5 level and the N10P2.5 level, and the P2.5 level and the N5P5 level showed differences (Table S2). The relative abundance of soil bacterial functional groups associated with aerobic nitrite oxidation significantly increased under N5, N10, and N10P2.5 treatments (Table S3), whereas the relative abundance of soil fungal functional groups did not significantly change under N and P additions.

Further analysis indicated that the composition of the soil fungal functional community was related to NH_4_^+^-N and N:P. The composition of the soil bacterial functional community was related to SR_plant_, SM, SOC, TP, TN, C:P, N:P, and SC (Table 2).

## 4. Discussion

### 4.1. Effects of N and P Additions on Soil Fungal and Bacterial Functional α-Diversity

Soil microbial functional diversity has great significance for the efficiency, resistance, and resilience of ecosystem processes [24]. It is necessary to predict the responses of soil microbial functional diversity to potential global changes. Our results showed that the direct effect caused by short-term N and P additions is a decrease in soil fungal functional α-diversity (Figure 2b,c), which was mainly mediated by soil available nitrogen as opposed to plant α-diversity or soil pH (Figure 3, Table 1). Short-term N and P additions caused a rapid increase in soil available nitrate and ammonium in soil [32]. Along with the addition time, the indirect influence factors on the plant community [37] and soil pH [13] would affect soil fungal functional diversity. Consistently, increasing evidence has shown that N and P additions altered soil fungal functional guilds in grasslands [6,37] and tropical forests [38]. However, there were no significant changes in soil fungal functional guilds in grassland and forest under N addition [20,39,40], which might be due to a deficiency of phosphorus.

Unlike the observations for soil fungi, no significant decrease in soil bacterial functional α-diversity in response to short-term N and P additions was observed in our study (Figure S6). Ding et al. [41] found that additional N reduced soil bacterial functional diversity in an agroecosystem, yet this trend was not evident in our study. The divergent results are potentially attributable to the different N supply levels—substantially higher in the agroecosystem (180 kg ha^−1^ yr^−1^) [41] than in our alpine steppe experiment (50, 100 kg ha^−1^ yr^−1^). Zhang and Yuan [42] reported that microbial functional diversity did not exhibit a significant decrease unless the N addition rate exceeded 40 kg ha^−1^ yr^−1^ in an alpine meadow on the Tibetan Plateau. However, the duration of their nitrogen addition experiment was longer than ours. There is a significant disparity in the time span between the two experiments. Meanwhile, corresponding evidence showed that no significant differences were observed in soil bacterial functional alpha diversity [22,38,43].

We found that the response of soil fungal functional α-diversity was faster than that of soil bacteria to short-term N and P additions in our study (Figure 2, Figure S6), which might be the result of the following reasons. Firstly, the soil bacterial community was more sensitive to the addition of organic fertilizers, while the soil fungal community was more sensitive to the addition of inorganic fertilizers [44]. Secondly, the inconsistent responses might be related to the differences in life-history strategies between soil fungi and bacteria. Compared to the soil bacterial community, the soil fungal community was an oligotrophic community [45], so they need to maintain a higher biomass carbon–nutrient ratio, lower nutrient requirements, and lower metabolic activity than bacteria [46,47]. Thirdly, the growth of soil bacteria had antagonistic effects on the growth of soil fungi [48]. Nutrient addition led to a decrease in F:B, which had been found in many studies [49,50,51,52,53]. Fourthly, soil fungi were more sensitive to nutrient addition than soil bacteria [54,55]. In general, the hyphae of soil fungi could extend deep into the soil, increasing the surface area for water and nutrient absorption, making it easier for them to obtain nutrients and resources, and thereby transfer nutrients and resources from abundant sites to limiting sites [56]. Nutrient addition could lead to the soil fungal community losing this advantage.

### 4.2. Effects of N and P Additions on Soil Fungal and Bacterial Functional Community Composition

We found that the bacterial functional groups of aerobic nitrite oxidation increased significantly under N5, N10, and N10P2.5 treatments, which was not in agreement with a previous study that aerobic nitrite oxidation significantly decreased under N32 for 17 years [20]. On the one hand, this was due to the shorter duration of the addition. On the other hand, the amount of N addition did not reach N saturation thresholds in alpine grasslands [57]. The changes in aerobic nitrite oxidation were mainly due to the increase in soil available nitrogen (Figure 1). Our result was consistent with the copiotrophic–oligotrophic hypothesis. At the same time, a global meta-analysis showed that N addition significantly increased aerobic nitrite oxidation [43], which was in consistent with our results. Nevertheless, N and P additions did not significantly change soil fungal functional groups, which was inconsistent with a previous study [5], maybe due to the short-term N and P additions in our study.

Soil bacterial and fungal functional β-diversity did not change under N and P additions, which was inconsistent with a global meta-analysis showing that N addition led to a more similar β-diversity; in particular, a greater negative N effect was observed in regions with lower soil total nitrogen and mean annual air temperature [43]. The soil nutrient content and mean annual air temperature on the TP were lower [31]. Lower soil total nitrogen sites were more susceptible to N-induced environmental filtering, which selected for competitive taxa [58] and led to functional convergence (lower β-diversity). In addition, temperature acted as a key abiotic filter [59]; in cold regions with low ambient N availability, this severe condition might bring about the highly diverse responses of different soil bacterial functional groups to N addition [43]. On the one hand, due to short-term nutrient addition, there was no direct effect on soil microbial functional β-diversity. On the other hand, the β-diversity mainly depended on the changes in soil pH and soil inorganic nitrogen [43]. However, there were no changes in soil pH and soil inorganic nitrogen (Figure 1).

## 5. Conclusions

Based on a short-term N and P addition experiment in an alpine steppe on the TP, we investigated the changes in soil microbial functional community structure. We found that N and P additions significantly decreased soil fungal functional α-diversity, but not soil bacteria, in one month. Soil available nitrogen, not the plant community, played a key role in changes in soil fungi under N and P additions. Moreover, the soil bacterial functional group, aerobic nitrite oxidation, significantly increased under combined N and P additions, but soil bacterial and fungal community composition and β-diversity did not change. Taken together, there were different responses of soil fungal and bacterial functional community structure to N and P additions. However, our single-season observations were preliminary and cannot capture interannual or seasonal dynamics. Future multi-year experiments with repeated seasonal sampling and process-rate measurements are needed to validate sustained effects.

## Figures and Tables

**Table 1:** Spearman correlation analysis of functional α-diversity of soil fungal communities with soil and plant variables.

Variables	Guilds	ACE	Chao1	Shannon	Simpson
Shannon_plant_	−0.03	0.16	0.09	0.00	−0.09
Simpson_plant_	−0.05	0.13	0.06	−0.02	−0.12
SR_plant_	−0.07	0.09	0.03	−0.03	−0.12
Pielou_plant_	−0.03	0.12	0.04	0.00	−0.08
NH_4_^+^-N	−0.08	−0.06	0.04	0.02	0.02
NO_3_^−^-N	−0.21	−0.14	−0.07	0.06	0.04
AN:AP	−0.16	−0.16	−0.07	0.06	0.05
NH_4_^+^-N:NO_3_^−^-N	0.21	0.11	0.16	−0.18	−0.16
pH	0.12	0.01	0.06	−0.11	−0.01
SM	−0.24	−0.14	−0.07	−0.24	−0.32 *
AP	−0.03	0.23	0.14	−0.14	−0.13
SOC	−0.03	0.10	0.11	−0.06	−0.09
TP	−0.29	−0.15	−0.09	−0.10	−0.15
TN	−0.11	−0.05	0.03	−0.12	−0.17
C:N	0.27	0.23	0.13	0.03	0.05
C:P	0.17	0.14	0.10	−0.09	−0.10
N:P	0.12	0.08	0.13	−0.16	−0.18
SC	0.05	0.02	0.04	−0.09	−0.11
CL	0.21	0.03	0.02	−0.09	−0.09
UA	0.11	−0.08	−0.04	−0.24	−0.19
CT	0.13	0.21	0.20	−0.26	−0.33 *
ALP	0.00	0.04	−0.07	0.03	−0.04

Note: * indicates a significant difference at the 0.05 level. Shannon_plant_: N and P coupling on Shannon index of plant community; Simpson_plant_: N and P coupling on Simpson index of plant community; SR_plant_: N and P coupling on species richness of plant community; Pielou_plant_: N and P coupling on pielou index of plant community. C:N: ratio of carbon to nitrogen; C:P: ratio of carbon to phosphorus; N:P: ratio of nitrogen to phosphorus; SC: soil sucrase; CL: soil cellulase; UA: soil urease; CT: soil catalase; ALP: soil alkaline phosphatase.

**Table 2:** Mantel tests between functional community composition of soil fungi and bacteria with soil and plant variables based on Bray–Curtis distance.

Variables	Fungi	Bacteria
Shannon_plant_	0.04	0.09
Simpson_plant_	0.06	0.04
SR_plant_	0.01	0.15 *
Pielou_plant_	0.16+	0.02
pH	0.01	−0.06
SM (%)	0.13+	0.49 **
AP (mg kg^−1^)	0.06	−0.15
NH_4_^+^-N (mg kg^−1^)	0.19 *	0.01
NO_3_^−^-N (mg kg^−1^)	−0.05	−0.01
SOC (%)	0.11	0.39 **
TP (%)	−0.03	0.23 **
TN (%)	0.11	0.46 **
C:N	−0.07	−0.02
C:P	0.14+	0.16 *
N:P	0.18 *	0.19 *
NH_4_^+^-N:NO_3_^−^-N	0.01	−0.06
AN:AP	−0.12	0.05
SC (mg g^−1^ d^−1^)	0.12	0.18 *
CL (mg g^−1^ 3 d^−1^)	0.03	0.04
UA (mg g^−1^ d^−1^)	−0.03	−0.03
CT (mL g^−1^ 20 min^−1^)	0.12+	0.11+
ALP (mg g^−1^ 2 h^−1^)	−0.02	0.16+

Note: * and ** indicate a significant difference at the 0.05 and 0.01 level, respectively.

## Data Availability

The raw data supporting the conclusions of this article will be made available by the authors on request.

## Supplementary Materials

The following supporting information can be downloaded at: https://www.mdpi.com/article/10.3390/biology15161332/s1, File S1: High-Throughput Sequencing; Figure S1: The study area; Figure S2: The plant community of the study site; Figure S3: Effects of N and P coupling on eight conventional soil physical and chemical properties; Figure S4: Effects of N and P coupling on plant α-diversity; Figure S5: Effects of N and P coupling on plant community composition based on Bray–Curtis distance: A nonmetric multidimensional analysis (NMDS); Figure S6: Effects of N and P coupling on soil bacterial function α-diversity in an alpine steppe; Figure S7: Relative contribution of observed soil and plant variables to soil bacterial function α-diversity; Table S1: Spearman correlation analysis of function α-diversity of soil bacterial communities with soil and plant variables; Figure S8: A nonmetric multidimensional analysis (NMDS) of N and P coupling on functional community composition of soil fungi (a) and bacteria (b) based on Bray–Curtis distance; Table S2: Significance test of N and P coupling on functional community composition of soil fungi and bacteria based on Bray–Curtis distance; Table S3: Duncan’s multiple comparison of soil bacterial functional groups based on LEFSe (different letters indicate significant differences between N and P additions, *p* < 0.05).
