# Supplementary material for: The Different Responses of the Soil Fungal and Bacterial Functional Community Structure to Short-Term N and P Additions in an Alpine Steppe on the Tibetan Plateau
Source: Biology (Basel). 2026 Aug 7;15(16):1332. doi: 10.3390/biology15161332 (PMC13509641; doi:10.3390/biology15161332)
Supplement: Supplementary file 1 [file biology-15-01332-s001.zip › biology-4450746-supplementary.pdf]

## File S1: High-Throughput Sequencing

We used 0.50 g soil to extract DNA. The concentration and purity were measured using the NanoDrop One (Thermo Fisher Scientific, MA, USA). The barcoded primers ITS1F (5′-CTTGGTCATTTAGAGGAAGTAA-3′) and ITS2R (5′-GCTGCGTTCTTCATCGATGC-3′) were used to amplify the ITS1 and ITS2 regions of the soil fungal community. The V4-V5 regions of the 16S rRNA gene were amplified by barcoded primers 515F (5′- GTGCCAGCMGCCGCGGTAA -3′) and 907R (5′-CCGTCAATTCMTTTRAGTTT -3′) for soil bacterial community. PCR reactions, containing 25 μL 2x Premix Taq (Takara Biotechnology, Dalian Co. Ltd., China), 1 μL each primer (10 μM) and 3 μL DNA (20 ng/μL) template in a volume of 50 μL, were amplified by thermocycling: 5 min at 94°C for initialization; 30 cycles of 30 s denaturation at 94°C, 30 s annealing at 52°C, and 30 s extension at 72°C; followed by 10 min final elongation at 72°C. The length and concentration of the PCR product were detected by 1% agarose gel electrophoresis. Samples with bright main strip between could be used for further experiments. PCR products were mixed in equidensity ratios according to the GeneTools Analysis Software (Version4.03.05.0, SynGene). Then, mixture of PCR products was purified with E.Z.N.A. Gel Extraction Kit (Omega, USA). Sequencing libraries were generated using NEBNext® Ultra™ II DNA Library Prep Kit for Illumina® (New England Biolabs, MA, USA). At last, the library was sequenced on an Illumina Nova6000 platform and 250 bp paired-end reads were generated.

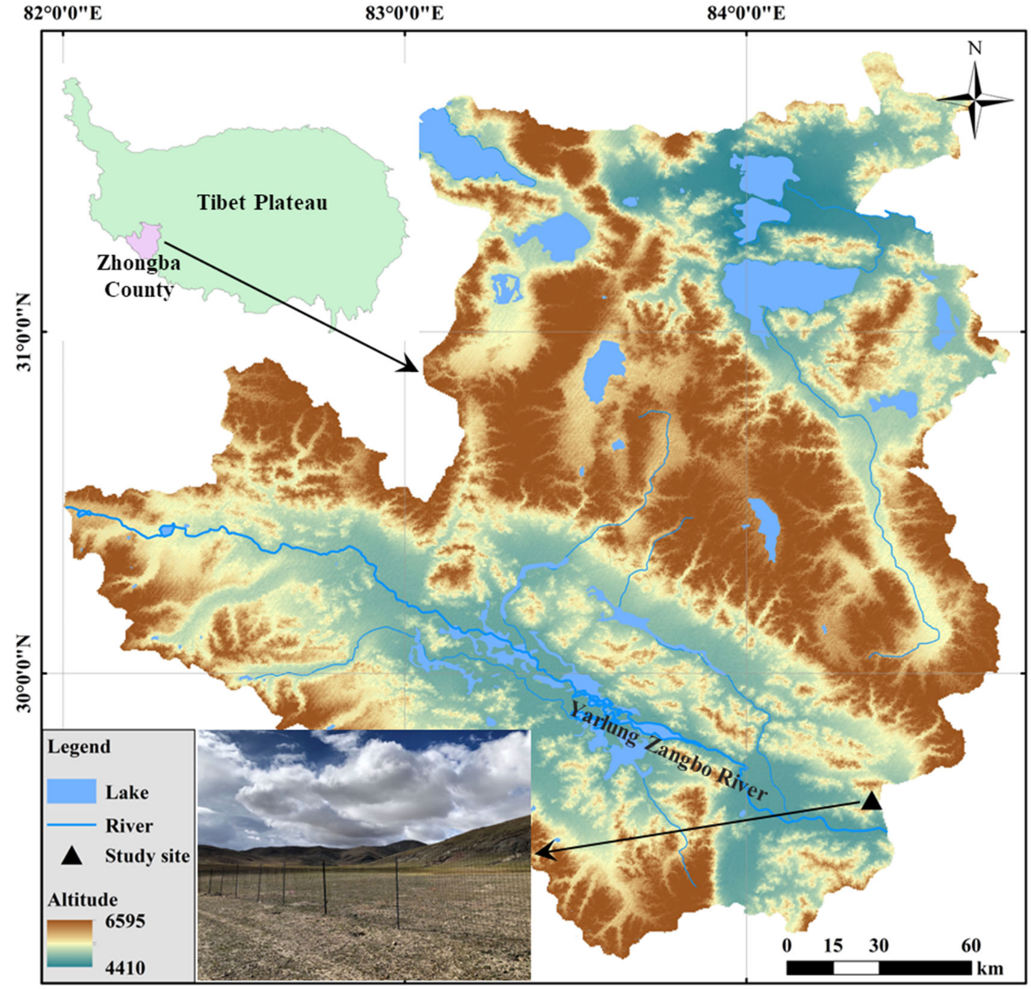


**Figure S1. The study area.**

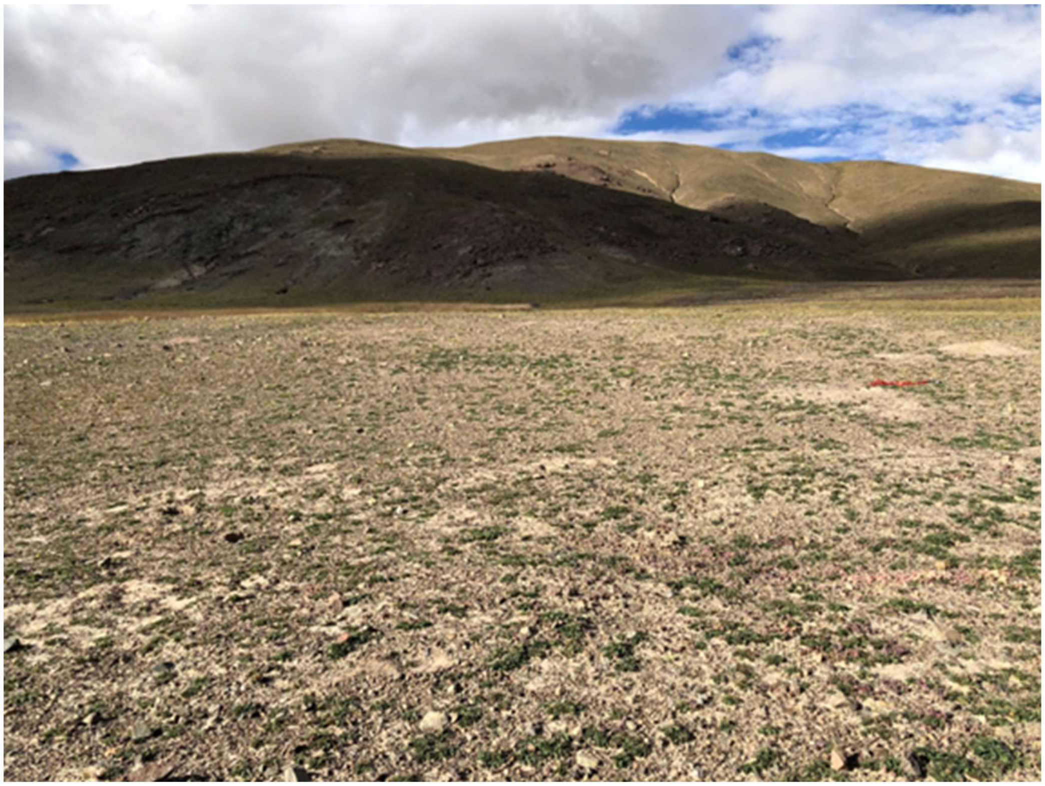

**Figure S2.** The plant community of the study site.

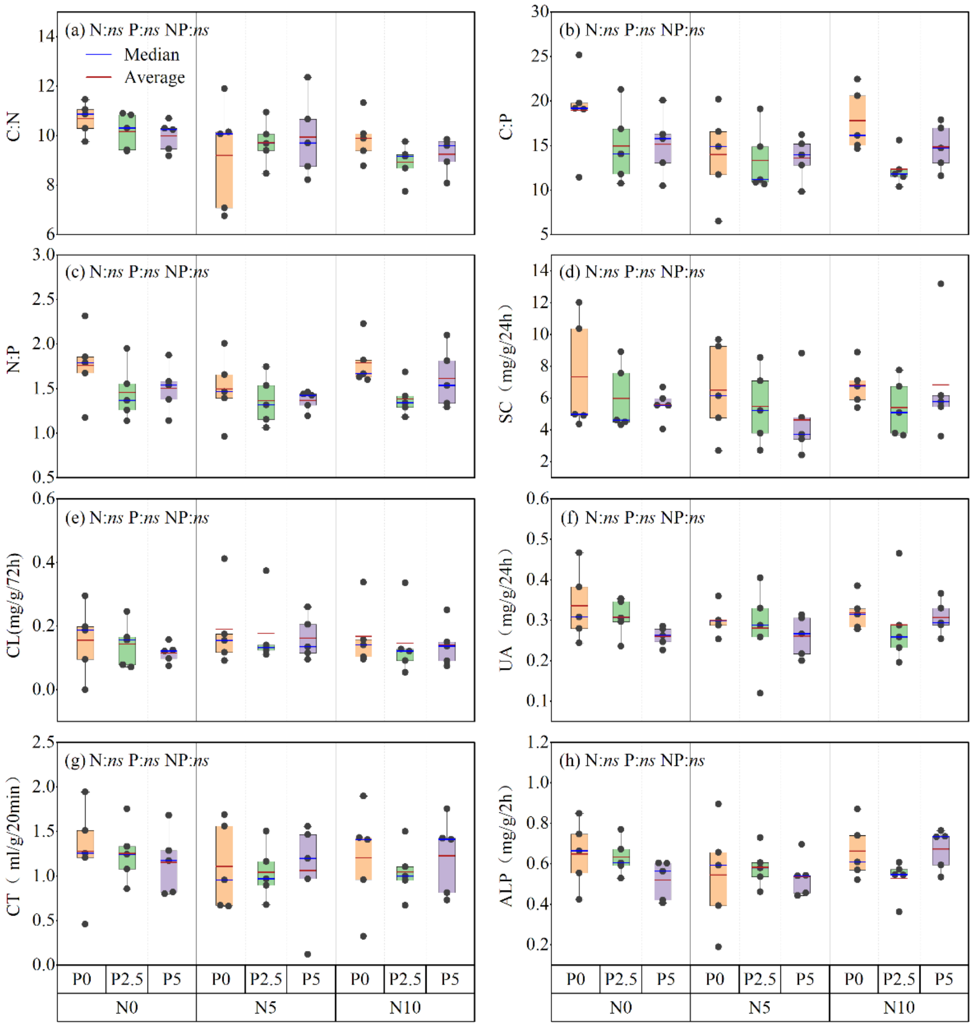


**Figure S3.** Effects of N and P coupling on eight soil conventional physical and chemical properties (mean ± SD, *n* = 5; *ns* indicates non-significant differences). (a) C: N: ratio of carbon to nitrogen; (b) C:P: ratio of carbon to phosphorus; (c) N:P: ratio of nitrogen to phosphorus; (d) SC: soil sucrase; (e) CL: soil cellulase; (f) UA: soil urease; (g) CT: soil catalase; (h) ALP: soil alkaline phosphatase.

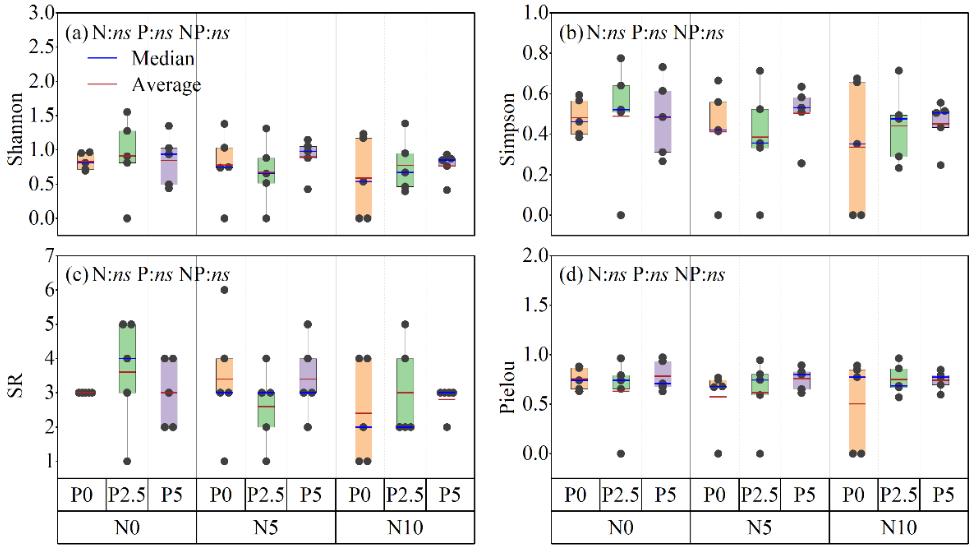


**Figure S4.** Effects of N and P coupling on plant α-diversity (mean ± SD, $n$ = 5; two-way ANOVA, *ns* indicates non-significant differences). (a) Shannon, (b) Simpson, (c) SR, (d) Pielou.

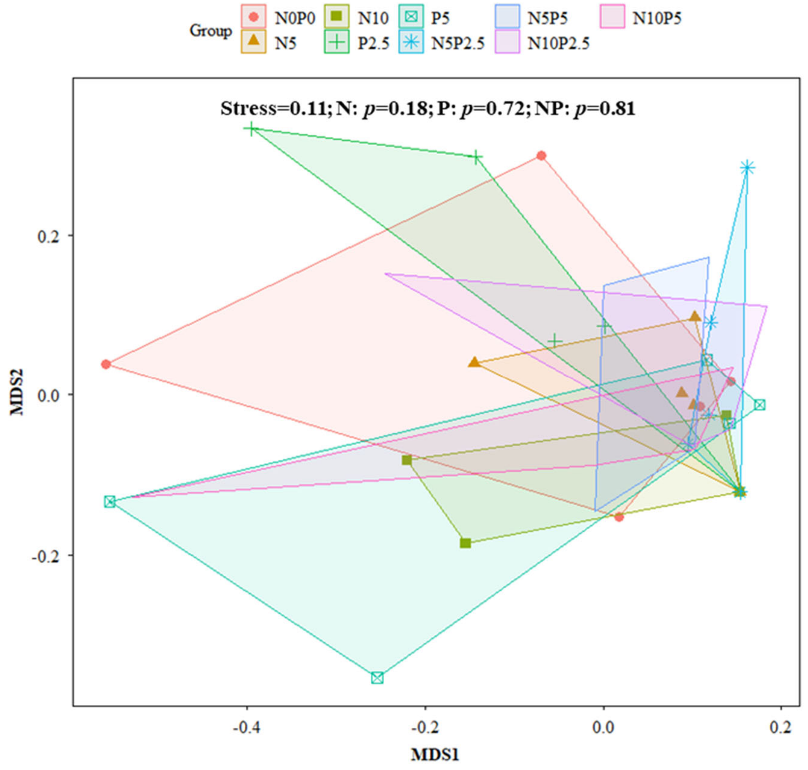


**Figure S5.** Effects of N and P coupling on plant community composition based on Bray–Curtis distance: A nonmetric multidimensional analysis (NMDS).

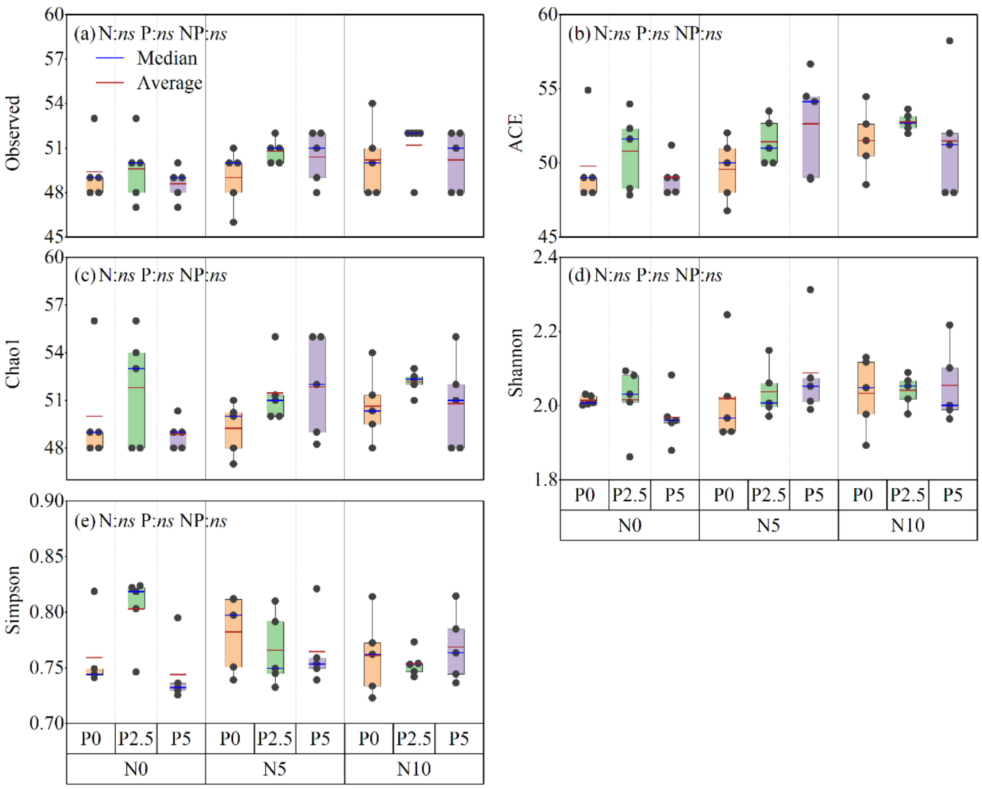


**Figure S6.** Effects of N and P coupling on soil bacterial function *α*-diversity in an alpine steppe (mean ± SD, $n$ = 5; two-way ANOVA, *ns* indicates non-significant differences). (a) Observed, (b) ACE, (c) Chao1, (d) Shannon, (e) Simpson.

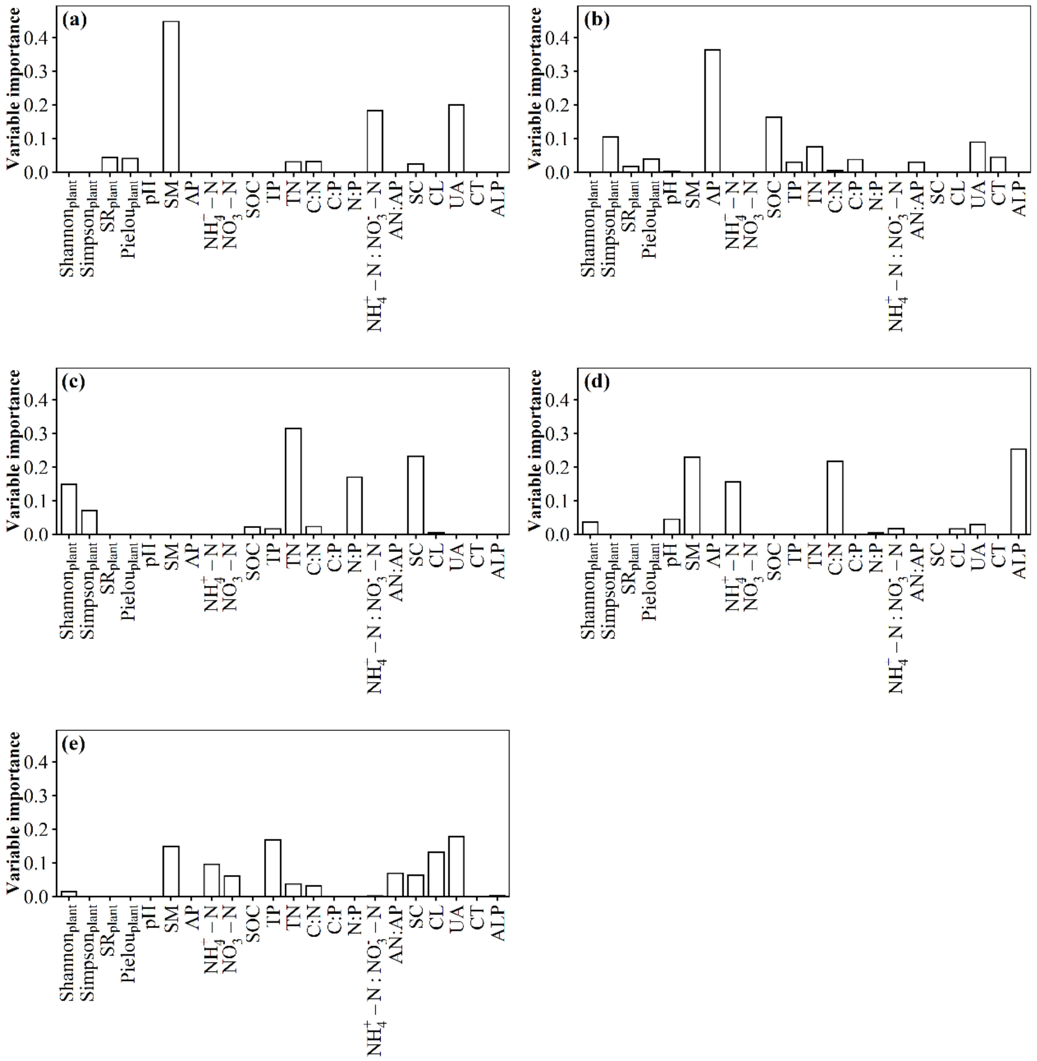


**Figure S7.** Relative contribution of observed soil and plant variables on soil bacterial function $\alpha$-diversity, including (a) Observed, (b) ACE, (c) Chao1, (d) Shannon, and (e) Simpson to N and P coupling in an alpine steppe.

**Table S1.** Spearman correlation analysis of function $\alpha$-diversity of soil bacterial communities with soil and plant variables.

| Variables | Observed | ACE | Chao1 | Shannon | Simpson |
|---|---|---|---|---|---|
| $Shannon_{plant}$ | 0.02 | 0.13 | 0.14 | **-0.35*** | -0.21 |
| $Simpson_{plant}$ | 0.06 | 0.15 | 0.15 | **-0.35*** | -0.21 |
| $SR_{plant}$ | -0.02 | 0.10 | 0.15 | -0.29 | -0.12 |
| $Pielou_{plant}$ | 0.08 | 0.13 | 0.07 | -0.26 | -0.25 |
| $NH_4^+$-N | **0.32*** | **0.43**** | **0.34*** | **0.31*** | 0.05 |
| $NO_3^-$-N | 0.29 | **0.36*** | 0.26 | 0.18 | 0.03 |
| AN: AP | **0.31*** | **0.41**** | **0.31*** | 0.24 | 0.09 |
| $NH_4^+$-N:$NO_3^-$-N | 0.08 | 0.16 | 0.18 | 0.18 | 0.00 |
| pH | 0.01 | 0.00 | 0.07 | 0.26 | 0.20 |
| SM | -0.29 | -0.29 | -0.27 | -0.15 | **0.48**** |
| AP | 0.09 | 0.15 | 0.08 | 0.18 | 0.01 |
| SOC | -0.25 | -0.23 | -0.28 | **-0.33*** | 0.02 |
| TP | **-0.32*** | **-0.33*** | **-0.36*** | -0.15 | **0.39**** |

| | | | | | |
|---|---|---|---|---|---|
| TN | -0.27 | -0.22 | -0.29 | -0.25 | 0.23 |
| C: N | -0.05 | -0.11 | -0.08 | -0.28 | -0.27 |
| C:P | -0.12 | -0.12 | -0.13 | **-0.32*** | -0.10 |
| N:P | -0.13 | -0.10 | -0.13 | -0.27 | 0.02 |
| SC | **-0.39**** | **-0.44**** | **-0.49**** | **-0.37*** | 0.06 |
| CL | 0.21 | 0.19 | 0.21 | 0.22 | 0.15 |
| UA | -0.04 | -0.13 | -0.13 | -0.18 | 0.09 |
| CT | -0.10 | -0.18 | -0.17 | -0.26 | 0.07 |
| ALP | -0.07 | -0.15 | -0.11 | **-0.35*** | 0.04 |

**Note:** Significant correlation are bolded.

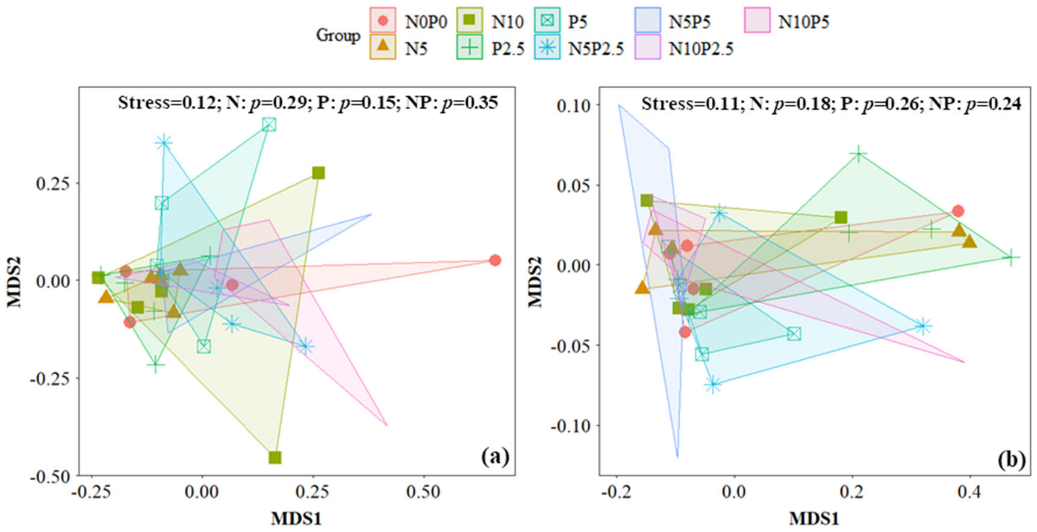


**Figure S8.** A nonmetric multidimensional analysis (NMDS) of N and P coupling on functional community composition of soil fungi (a) and bacteria (b) based on Bray–Curtis distance.

**Table S2.** Significance test of N and P coupling on functional community composition of soil fungi and bacteria based on Bray–Curtis distance.

| | | | N0P0 | N5 | N10 | P2.5 | P5 | N5P2.5 | N10P2.5 | N5P5 |
|---|---|---|---|---|---|---|---|---|---|---|
| Fungi | Adonis2 *p* value | N5 | 0.77 | | | | | | | |
| | | N10 | 0.84 | 0.25 | | | | | | |
| | | P2.5 | 0.68 | 0.35 | 0.59 | | | | | |
| | | P5 | 0.92 | 0.70 | 0.70 | 0.17 | | | | |
| | | N5P2.5 | 0.31 | **0.03** | 0.22 | 0.20 | 0.22 | | | |
| | | N10P2.5 | 0.62 | 0.35 | 0.35 | 0.16 | 0.87 | 0.13 | | |
| | | N5P5 | 0.88 | 0.18 | 0.64 | 0.26 | 0.76 | 0.73 | 0.83 | |
| | | N10P5 | 0.21 | **0.01** | 0.29 | **0.02** | 0.40 | 0.16 | 0.33 | 0.52 |
| | | | N0P0 | N5 | N10 | P2.5 | P5 | N5P2.5 | N10P2.5 | N5P5 |
| Bacteria | Adonis2 *p* value | N5 | 0.63 | | | | | | | |
| | | N10 | 0.98 | 0.39 | | | | | | |
| | | P2.5 | 0.15 | 0.38 | **0.03** | | | | | |
| | | P5 | 0.81 | 0.22 | 0.64 | **0.03** | | | | |
| | | N5P2.5 | 0.76 | 0.36 | 0.62 | 0.13 | 0.74 | | | |
| | | N10P2.5 | 0.43 | 0.40 | 0.42 | **0.02** | 0.13 | 0.08 | | |
| | | N5P5 | 0.48 | 0.34 | 0.31 | **0.02** | 0.29 | 0.14 | 0.81 | |
| | | N10P5 | 0.87 | 0.91 | 0.92 | 0.14 | 0.58 | 0.50 | 0.96 | 0.90 |

**Table S3.** Duncan Multiple Comparison of soil bacterial functional groups based on LEFSe (different letters indicate significant differences between N and P addition additions, $p < 0.05$).

| | N0P0 | N5 | N10 | P2.5 | P5 | N5P2.5 | N10P2.5 | N5P5 | N10P5 |
|---|---|---|---|---|---|---|---|---|---|
| methanotrophy | 63.80±35.1 a | 43.80±24.54 a | 41.00±31.26 a | 50.20±26.74 a | 73.00±40.42 a | 90.00±52.00 a | 37.60±14.93 a | 82.60±51.54 a | 62.00±67.22 a |
| methanol_oxidation | 3138.60±6046.13 a | 6065.60±8023.46 a | 1686.00±2867.75 a | 9632.80±7144.19 a | 1082.40±1623.32 a | 2634.00±4407.34 a | 515.20±590.46 a | 449.60±250.44 a | 3366.80±7040.99 a |
| methylotrophy | 3139.00±6045.89 a | 6066.80±8023.68 a | 1690.40±2868.48 a | 9633.20±7144.09 a | 1086.00±1623.22 a | 2635.20±4407.21 a | 516.80±590.18 a | 451.00±250.31 a | 3371.60±7041.12 a |
| aerobic_ammonia_oxidation | 590.40±133.98 a | 635.00±53.63 a | 664.20±137.63 a | 610.40±61.61 a | 519.60±108.25 a | 620.80±146.74 a | 601.40±66.44 a | 597.40±81.32 a | 677.20±254.89 a |
| **aerobic_nitrite_oxidation** | **223.00±45.37 bc** | **351.40±118.26 a** | **355.20±99.92 a** | **180.20±22.02 c** | **207.80±142.16 bc** | **261.20±106.89 abc** | **348.40±27.84 a** | **234.80±72.21 abc** | **317.00±53.49 ab** |
| nitrification | 813.40±173.00 a | 986.40±164.14 a | 1019.40±118.52 a | 790.60±46.32 a | 727.40±242.46 a | 882.00±189.01 a | 949.80±75.47 a | 832.20±152.76 a | 994.20±276.64 a |
| sulfate_respiration | 20.00±13.77 a | 17.60±13.32 a | 29.80±15.19 a | 14.40±5.41 a | 12.20±10.96 a | 16.80±13.20 a | 16.20±7.29 a | 17.20±14.36 a | 29.20±27.23 a |
| sulfur_respiration | 0.00±0.00 a | 0.00±0.00 a | 0.00±0.00 a | 0.60±1.34 a | 0.00±0.00 a | 0.00±0.00 a | 0.20±0.45 a | 0.00±0.00 a | 0.00±0.00 a |
| respiration_of_sulfur_compounds | 20.00±13.77 a | 17.60±13.32 a | 29.80±15.19 a | 15.00±5.52 a | 12.20±10.96 a | 16.80±13.20 a | 16.40±7.64 a | 17.20±14.36 a | 29.20±27.23 a |
| nitrate_denitrification | 107.40±39.87 a | 78.60±12.30 a | 103.00±30.88 a | 92.00±14.02 a | 86.60±23.61 a | 91.60±39.24 a | 90.40±10.55 a | 68.60±26.39 a | 92.80±35.44 a |
| nitrite_denitrification | 107.40±39.87 a | 78.60±12.30 a | 103.00±30.88 a | 92.00±14.02 a | 86.60±23.61 a | 91.60±39.24 a | 90.40±10.55 a | 68.60±26.39 a | 92.80±35.44 a |
| nitrous_oxide_denitrification | 107.40±39.87 a | 78.60±12.30 a | 103.00±30.88 a | 92.00±14.02 a | 86.60±23.61 a | 91.60±39.24 a | 90.40±10.55 a | 68.60±26.39 a | 92.80±35.44 a |
| denitrification | 107.40±39.87 a | 78.60±12.30 a | 103.00±30.88 a | 92.00±14.02 a | 86.60±23.61 a | 91.60±39.24 a | 90.40±10.55 a | 68.60±26.39 a | 92.80±35.44 a |
| chitinolysis | 22.80±11.65 a | 17.60±10.83 a | 47.80±28.66 a | 50.40±67.13 a | 18.80±5.26 a | 23.40±23.86 a | 21.00±8.28 a | 34.80±21.36 a | 32.40±18.99 a |
| dark_hydrogen_oxidation | 1013.20±187.27 a | 955.80±199.35 a | 1062.00±261.80 a | 927.40±207.70 a | 904.80±482.04 a | 851.80±378.25 a | 897.20±129.13 a | 699.40±326.86 a | 944.60±295.97 a |
| nitrogen_fixation | 613.20±136.63 a | 663.40±186.65 a | 604.00±255.38 a | 617.00±156.96 a | 621.00±219.45 a | 565.80±158.17 a | 579.20±137.09 a | 830.60±821.79 a | 605.20±129.46 a |
| nitrite_respiration | 107.40±39.87 a | 78.60±12.30 a | 103.00±30.88 a | 92.00±14.02 a | 86.60±23.61 a | 91.60±39.24 a | 90.40±10.55 a | 68.60±26.39 a | 92.80±35.44 a |
| cellulolysis | 40.60±28.34 a | 19.00±8.25 a | 30.00±12.35 a | 39.00±61.09 a | 36.60±13.74 a | 58.00±28.31 a | 28.20±16.02 a | 48.80±29.34 a | 41.60±30.36 a |
| xylanolysis | 0.00±0.00 a | 0.00±0.00 a | 0.20±0.45 a | 3.60±8.05 a | 0.00±0.00 a | 0.40±0.89 a | 0.40±0.89 a | 0.00±0.00 a | 0.60±1.34 a |
| dark_thiosulfate_oxidation | 16.00±9.82 a | 7.00±3.39 a | 10.00±13.77 a | 12.00±10.75 a | 20.20±10.18 a | 16.20±17.31 a | 6.40±4.51 a | 9.20±4.76 a | 8.80±6.26 a |
| dark_oxidation_of_sulfur_compounds | 19.20±10.03 a | 13.40±11.61 a | 13.20±14.13 a | 15.40±8.88 a | 22.60±11.24 a | 21.20±21.1 a | 8.80±3.27 a | 12.20±5.45 a | 13.40±8.08 a |
| manganese_oxidation | 103.20±53.50 a | 160.60±94.11 a | 250.80±194.89 a | 86.20±53.35 a | 125.80±38.30 a | 353.40±444.55 a | 284.80±260.78 a | 212.00±242.04 a | 230.40±245.98 a |
| manganese_respiration | 3.80±6.10 a | 1.00±1.41 a | 1.20±1.30 a | 1.60±2.51 a | 1.80±2.68 a | 4.80±2.95 a | 2.80±3.11 a | 10.20±11.69 a | 4.80±7.12 a |
| ligninolysis | 3.40±2.30 a | 2.60±2.07 a | 3.40±4.34 a | 4.40±4.98 a | 6.20±4.66 a | 4.08±2.68 a | 2.40±2.70 a | 7.60±7.16 a | 7.40±11.63 a |
| fermentation | 288.40±81.23 a | 312.20±170.12 a | 338.60±101.93 a | 220.80±81.32 a | 334.80±126.93 a | 480.80±307.59 a | 305.20±70.49 a | 415.40±142.04 a | 313.80±76.79 a |
| aerobic_chemoheterotrophy | 10532.60±1552.63 a | 9334.20±782.61 a | 11024.40±1122.09 a | 9454.20±1748.32 a | 11770.40±1078.51 a | 12120.40±1717.88 a | 10195.40±1038.93 a | 10123.60±1930.16 a | 10280.40±1574.61 a |

| | N0P0 | N5 | N10 | P2.5 | P5 | N5P2.5 | N10P2.5 | N5P5 | N10P5 |
|---|---|---|---|---|---|---|---|---|---|
| invertebrate_parasites | 0.60±0.89 a | 0.00±0.00 a | 1.20±0.84 a | 0.20±0.45 a | 0.80±1.30 a | 0.60±0.89 a | 0.60±0.55 a | 0.40±0.55 a | 0.00±0.00 a |
| human_pathogens_nosocomia | 0.00±0.00 a | 0.20±0.45 a | 0.00±0.00 a | 0.00±0.00 a | 0.00±0.00 a | 0.00±0.00 a | 0.00±0.00 a | 0.00±0.00 a | 0.00±0.00 a |
| human_pathogens_all | 16.60±5.03 a | 10.80±2.39 a | 19.20±11.67 a | 10.80±6.80 a | 19.80±7.56 a | 17.00±12.86 a | 11.00±2.55 a | 20.20±11.17 a | 14.60±4.93 a |
| human_gut | 13.60±26.53 a | 2.00±1.22 a | 1.60±1.82 a | 1.20±1.64 a | 1.80±3.03 a | 1.00±1.22 a | 15.60±20.89 a | 1.20±0.84 a | 3.60±2.70 a |
| human_associated | 30.20±23.61 a | 12.80±3.11 a | 20.80±11.17 a | 12.00±5.70 a | 21.60±8.44 a | 18.00±12.02 a | 26.60±22.35 a | 21.40±10.88 a | 18.20±5.89 a |
| mammal_gut | 13.60±26.53 a | 2.00±1.22 a | 1.60±1.82 a | 1.20±1.64 a | 1.80±3.03 a | 1.00±1.22 a | 15.60±20.89 a | 1.20±0.84 a | 3.60±2.70 a |
| animal_parasites_or_symbionts | 33.60±23.78 a | 16.20±5.36 a | 26.40±8.82 a | 18.00±6.75 a | 25.60±8.59 a | 21.60±12.90 a | 35.20±26.78 a | 26.60±12.62 a | 22.20±4.87 a |
| aromatic_hydrocarbon_degradation | 15.80±12.32 a | 11.80±11.26 a | 20.60±11.46 a | 19.20±18.49 a | 26.00±22.39 a | 31.80±39.33 a | 11.00±6.96 a | 22.00±17.68 a | 30.80±25.68 a |
| aromatic_compound_degradation | 263.40±129.02 bcd | 238.80±114.69 bcd | 219.80±77.43 cd | 213.80±126.45 d | 435.80±125.33 a | 371.00±65.54 ab | 303.00±89.96 abcd | 363.60±89.63 abc | 296.40±77.37 abcd |
| aliphatic_non_methane_hydrocarbon_degradation | 15.80±12.32 a | 11.80±11.26 a | 20.60±11.46 a | 19.20±18.49 a | 26.00±22.39 a | 31.80±39.33 a | 11.00±6.96 a | 22.00±17.68 a | 30.80±25.68 a |
| hydrocarbon_degradation | 79.60±35.25 a | 55.60±16.59 a | 61.60±41.51 a | 69.40±32.52 a | 99.00±51.48 a | 121.80±83.69 a | 48.60±18.65 a | 104.60±62.75 a | 92.80±87.04 a |
| iron_respiration | 6.40±7.77 a | 2.20±2.28 a | 2.80±2.95 a | 3.40±3.21 a | 2.60±3.58 a | 6.20±3.35 a | 4.40±3.78 a | 13.60±15.22 a | 7.20±9.93 a |
| nitrate_respiration | 115.20±39.81 a | 90.20±12.93 a | 110.60±31.57 a | 99.20±16.33 a | 94.20±31.07 a | 101.00±42.36 a | 100.80±8.53 a | 77.20±33.48 a | 102.00±40.74 a |
| nitrate_reduction | 995.00±468.71 a | 887.60±212.43 a | 868.60±289.54 a | 915.40±325.96 a | 1127.60±675.96 a | 1055.60±361.58 a | 1082.80±135.63 a | 966.40±272.18 a | 1015.00±352.58 a |
| nitrogen_respiration | 115.20±39.81 a | 90.20±12.93 a | 110.60±31.57 a | 99.20±16.33 a | 94.20±31.07 a | 101.00±42.36 a | 100.80±8.53 a | 77.20±33.48 a | 102.00±40.74 a |
| fumarate_respiration | 3.80±6.10 a | 1.00±1.41 a | 1.20±1.30 a | 1.60±2.51 a | 1.80±2.68 a | 4.80±2.95 a | 2.80±3.11 a | 10.20±11.69 a | 4.80±7.12 a |
| intracellular_parasites | 39.00±21.07 a | 24.40±13.90 a | 29.80±15.42 a | 20.00±13.93 a | 33.60±11.67 a | 36.60±23.20 a | 27.60±9.15 a | 27.80±17.27 a | 33.20±26.66 a |
| predatory_or_exoparasitic | 166.60±35.03 a | 141.40±47.46 a | 162.40±55.88 a | 127.60±38.77 a | 135.80±30.99 a | 167.80±54.45 a | 153.60±35.06 a | 151.60±58.03 a | 166.80±41.24 a |
| nonphotosynthetic_cyanobacteria | 19.60±6.84 a | 20.20±7.43 a | 20.40±6.80 a | 16.60±5.94 a | 23.40±7.70 a | 21.40±11.59 a | 17.60±5.59 a | 17.40±4.62 a | 22.20±9.73 a |
| photosynthetic_cyanobacteria | 136.80±133.02 a | 183.80±301.49 a | 95.20±101.68 a | 141.00±123.62 a | 126.60±71.01 a | 150.60±87.67 a | 96.40±79.29 a | 480.40±833.31 a | 112.20±112.19 a |
| anoxygenic_photoautotrophy_S_oxidizing | 106.20±40.16 a | 75.80±15.72 a | 102.00±30.55 a | 91.40±14.22 a | 84.60±24.76 a | 90.20±39.18 a | 89.40±10.41 a | 62.20±25.61 a | 90.80±34.82 a |
| anoxygenic_photoautotrophy | 106.20±40.16 a | 75.80±15.72 a | 102.00±30.55 a | 91.40±14.22 a | 84.60±24.76 a | 90.20±39.18 a | 89.40±10.41 a | 62.20±25.61 a | 90.80±34.82 a |
| oxygenic_photoautotrophy | 136.80±133.02 a | 183.80±301.49 a | 95.20±101.68 a | 141.00±123.62 a | 126.60±71.01 a | 150.60±87.67 a | 96.40±79.29 a | 480.40±833.31 a | 112.20±112.19 a |
| photoautotrophy | 243.00±131.90 a | 259.60±290.83 a | 197.20±116.38 a | 232.40±113.50 a | 211.20±52.82 a | 240.80±77.32 a | 185.80±82.00 a | 542.60±842.24 a | 203.00±107.94 a |
| photoheterotrophy | 193.80±55.71 a | 156.80±52.57 a | 191.40±60.48 a | 172.40±27.13 a | 138.80±27.38 a | 187.80±96.04 a | 162.20±20.87 a | 127.20±48.41 a | 187.00±53.33 a |
| phototrophy | 330.06±156.54 a | 340.60±265.36 a | 286.60±139.99 a | 313.40±115.48 a | 265.40±55.83 a | 338.40±76.38 a | 258.60±87.13 a | 607.60±838.96 a | 299.20±101.93 a |
| ureolysis | 263.60±87.58 a | 198.40±36.2 a | 251.40±71.30 a | 281.80±240.63 a | 345.00±198.69 a | 299.00±135.36 a | 229.20±82.84 a | 320.00±95.28 a | 265.20±56.08 a |
| reductive_acetogenesis | 0.60±1.34 a | 0.00±0.00 a | 1.60±3.05 a | 0.20±0.45 a | 0.60±1.34 a | 0.00±0.00 a | 0.80±1.30 a | 0.00±0.00 a | 0.20±0.45 a |
| chemoheterotrophy | 13851.80±4673.84 a | 15594.00±7355.05 a | 12930.40±2943.34 a | 19261.00±5533.84 a | 13027.60±2317.24 a | 15099.00±5512.12 a | 10896.60±1263.68 a | 10781.60±1973.88 a | 13855.20±8389.75 a |
| anaerobic_chemoheterotrophy | 3319.20±6000.99 a | 6259.80±7929.42 a | 1906.00±2863.85 a | 9806.80±7093.39 a | 1257.20±1643.37 a | 2978.60±4394.63 a | 701.20±578.82 a | 658.00±262.70 a | 3574.80±7004.56 a |
